# The regulatory effect of zinc on the association between periodontitis and atherosclerotic cardiovascular disease: a cross-sectional study based on the National Health and Nutrition Examination Survey

**DOI:** 10.1186/s12903-024-04473-6

**Published:** 2024-06-18

**Authors:** Xiuxiu Yang, Maoyu Luo, Yao Jiang

**Affiliations:** Department of Stomatology, Ziyang Central Hospital, No.66 Rende Western Road, Yanjiang District, Ziyang, 641300 P.R. China

**Keywords:** Zinc intake, Periodontitis, Atherosclerotic cardiovascular disease, Cross-sectional study

## Abstract

**Background:**

Zinc has been proven to be effective against periodontitis, and also reported to reduce the risk of cardiovascular diseases (CVD). This study aims to explore the regulatory effect of zinc intake on the association between periodontitis and atherosclerotic cardiovascular disease (ASCVD).

**Methods:**

This was a cross-sectional study based on the National Health and Nutrition Examination Survey (NHANES). Logistic regression model was used to explore the association between zinc-RDA or periodontitis and 10-year ASCVD risk ≥ 20%, and results were shown as odds ratio (OR) and 95% confidence interval (95% CI). The regulatory effect of zinc intake on the association between periodontitis and 10-year ASCVD risk ≥ 20% was also assessed using logistic regression model. Subgroup analysis was performed based on age, gender, obesity, education level, lipid-lowering therapy, and dental floss.

**Results:**

6,075 patients were finally included for analysis. Zinc intake reaching the recommended level (OR = 0.82, 95%CI: 0.69–0.98) and periodontitis (OR = 2.47, 95%CI: 2.04-3.00) were found to be associated with 0.82-fold and 2.47-fold odds of 10-year ASCVD risk ≥ 20%, respectively. In addition, we found that the odds of 10-year ASCVD risk ≥ 20% was lower in patients with zinc intake reaching the recommended level than those without [OR (95%CI): 2.25 (1.81–2.80) vs. 2.72 (2.05–3.62)]. The similar regulatory effect was found in patients with age ≥ 60 years and < 60 years, in male and female, with or without obesity, in different education levels, with or without lipid lowering therapy, and with or without use of dental floss (all *P* < 0.05).

**Conclusions:**

This study found the regulatory effect of adequate zinc intake on the association between periodontitis and ASCVD, providing guidance for periodontitis patients to decrease the risk of ASCVD.

**Supplementary Information:**

The online version contains supplementary material available at 10.1186/s12903-024-04473-6.

## Background

Periodontitis is an inflammatory disease associated with the accumulation of dental plaque, and characterized by progressive destruction of the teeth-supporting apparatus [1]. In the United States, periodontitis affects more than 40% of adults, and is a leading cause for tooth loss [1, 2]. In addition to the impact on oral health, periodontitis may also be associated with an increased risk of atherosclerotic cardiovascular disease (ASCVD) [3, 4]. Therefore, paying attention to the association between periodontitis and ASCVD and identifying modifiable influencing factors is of great significance in reducing the disease burden of periodontitis.

Periodontitis could influence ASCVD according to periodontal pathogens directly invading endothelial cells, the inflammation and oxidative stress [5]. Zinc, a nutritional trace element, could regulate immune response and have antioxidant/anti-inflammatory activities [6]. Zinc can retard the oxidative processes by inducing the expression of metallothioneins, and play anti-inflammatory role by increasing the gene expression of proteins with anti-inflammatory properties [6]. Zinc has been proven to be effective against oral health problems including periodontitis, and also used in oral health care products to control and inhibit the formation of dental plaque and dental calculus [7]. In general population, supplementing zinc can decrease the risk of atherosclerosis and prevent the incidence of cardiovascular diseases (CVD) [8]. However, the modulatory effect of zinc on the association between periodontitis and ASCVD has not been reported.

The ASCVD risk score recommended by the American College of Cardiology (ACC) and American Heart Association (AHA) is a common method to estimate the 10-year ASCVD risk to guide decisions about preventive interventions [9]. In this study, we aimed to explore the association between periodontitis and ASCVD assessed by 10-year ASCVD risk score, and further explore the modulatory effect of zinc on the association between periodontitis and ASCVD, providing a certain basis for the management of periodontal health and the prevention of ASCVD.

## Methods

### Study design and data source

This was a cross-sectional study and data were extracted from the National Health and Nutrition Examination Survey (NHANES) (https://wwwn.cdc.gov/Nchs/Nhanes/). NHANES is a program designed to evaluate the health and nutritional status of adults and children in the United States. The survey examined a nationally representative sample of about 5,000 persons every year using a stratified multistage sampling design with a weighting scheme, and combined interviews and physical examinations. The requirement of ethical approval for this was waived by the Institutional Review Board of the Ziyang Central Hospital, because the data was accessed from NHANES (a publicly available database). The need for written informed consent was waived by the Institutional Review Board of the Ziyang Central Hospital due to retrospective nature of the study. All methods were performed in accordance with the relevant guidelines and regulations.

### Study population

Periodontitis patients aged 40–79 years old from NHANES database in 2009–2014 were included in this study. The inclusions were as follows: (1) age ≥ 40 years old and < 80 years old; (2) having examinations information on periodontitis; (3) having data on zinc intake; and (4) in the database from 2019 to 2014. Exclusion criteria were as follows: (1) less than two teeth (excluding third molars); (2) missing data on the intake of zinc; (3) with extreme total energy intakes (energy intake < 500 kcal or ≥ 8000 kcal in male or < 500 kcal or ≥ 5000 kcal in female) [10]; (4) with history of CVD; (5) missing data on smoking; (6) missing data on body mass index (BMI); and (7) missing data on the assessment of ASCVD.

Periodontitis was defined based on attachment loss and pocket depth, and divided into three categories (mild, moderate, and severe) [11, 12]. Mild periodontitis was defined as ≥ 2 interproximal sites with attachment loss ≥ 3 mm, and ≥ 2 interproximal sites with pocket depth ≥ 4 mm (not on the same tooth) or one site with pocket depth ≥ 5 mm. Moderate periodontitis was defined as ≥ 2 interproximal sites with attachment loss ≥ 4 mm (not on the same tooth), or ≥ 2 interproximal sites with pocket depth ≥ 5 mm (not on the same tooth). Severe periodontitis was defined as ≥ 2 interproximal sites with attachment loss ≥ 6 mm (not on the same tooth) and ≥ 1 interproximal site with pocket depth ≥ 5 mm [11, 12]. No periodontitis was defined as no evidence of mild, moderate, and severe periodontitis [11, 12].

The intake of zinc was obtained via 24-hour dietary recall interview, and included the sum of food, beverages, and dietary supplements. The 24-hour dietary recall interviews were conducted twice, and requested participants to recall all the food and beverages consumed in the past 24 h [13]. The first interview was performed in-person in the Mobile Examination Center (MEC), and the second interview was conducted by telephone 3 to 10 days later [13]. In this study, we used the intake of zinc reported in the first interview. The intake of zinc from dietary supplements was obtained through questionnaire, and someone who answered ‘yes’ when be asked whether used any dietary supplements in the past 30 days was further asked about the product name, frequency, duration, and serving form [14]. According to the recommended dietary allowances (RDA) (https://health.gov/sites/default/files/2019-09/2015-2020_Dietary_Guidelines.pdf), zinc intake was divided into two groups: zinc-RDA (yes) and zinc-RDA (no).

Participants answered ‘yes’ when be asked whether they had ever been diagnosed with coronary heart disease, angina, heart failure, heart attack, or stroke by a doctor or self-reported the use of cardiovascular drugs was defined as having CVD [15].

The ASCVD risk score was utilized to predict the 10-year ASCVD risk in individuals based on the age, sex, race, cholesterol levels, blood pressure, medication use, diabetic status, and smoking status of the participants [9]. In our study, patients were divided into 10-year ASCVD risk < 20% (low to intermediate risk) and 10-year ASCVD risk ≥ 20% (high risk) [9].

### Data extraction

Data were extracted based on demographics (age, gender, race, educational level, poverty-to-income ratio [PIR], marital status), physical examination [BMI], living habits (smoking and drinking status, physical activity, sedentary time), diabetes, medicine use (diabetes drugs, hypertension drugs, lipid-lowering therapy, nonsteroidal drugs, anti-infective drugs), laboratory values (total cholesterol [TC], high density lipoprotein cholesterol [HDL-C], glycated hemoglobin [HbAlc], serum vitamin D, white blood cell count [WBC]), oral health (decayed teeth, oral hygiene, dental floss), and dietary intake (total energy, carbohydrate, vitamin C).

BMI was calculated as body weight (kg)/height (m)^2^, and divided into underweight (< 18.5 kg/m^2^), normal weight (18.5–24.9 kg/m^2^), overweight (25–29.9 kg/m^2^), and obese (≥ 30 kg/m^2^) [16].

Physical activity was evaluated via converting into energy expenditure, which was calculated as the recommended metabolic equivalent of task (MET) × exercise time (min). Physical activity was divided into < 450 MET*min/week, ≥ 450 MET*min/week, and unknown [17].

Diabetes was determined according to one of the following conditions: (1) HbA1c ≥ 6.5%; (2) fasting blood glucose (FBG) ≥ 126 mg/dL; (3) serum glucose at 2 h following a 75 g glucose load (OGTT) ≥ 200 mg/dL; (4) self-reported diagnosis of diabetes; (5) self-reported use of insulin or other diabetes medication [18].

### Statistical analysis

Data used in this study were weighted using sample weights from NHANES. The continuous variables were expressed as mean (standard error) (S.E), with t test for comparison between two groups and analysis of variance for comparison more than two groups. The categorical variables were expressed as number and percentage [n (%)], with chi-squared test for comparison between two groups. Variables with missing value ≤ 5% were processed using random forest imputation method, and variables with missing value > 5% were deleted [19]. Sensitivity analysis was performed to avoid some biases caused by imputation.

Univariate logistic regression model was used to select potential confounders, and variables with statistical difference (*P* < 0.05) were determined as confounders. The association between zinc-RDA or periodontitis and 10-year ASCVD risk ≥ 20% was assessed using univariate and multivariate logistic regression model, with results shown as odds ratio (OR) and 95% confidence interval (95% CI). Model 1 was unadjusted model, model 2 adjusted selected confounders, and model 3 adjusted zinc-RDA or periodontitis based on model 2. The modulatory effect of zinc on the association between periodontitis and 10-year ASCVD risk ≥ 20% was also assessed using univariate and multivariate logistic regression model. Subgroup analysis based on age, gender, obesity, education level, lipid-lowering therapy, and dental floss was performed. Statistical analysis was performed using Python 3.9.12 (Python Software Foundation, Delaware, USA) and R (version 4.3.1, R Foundation for Statistical Computing, Vienna, Austria, 2018). *P* < 0.05 was regarded as statistical significance.

## Results

### Selection and characteristics of patients

**Fig. 1:**
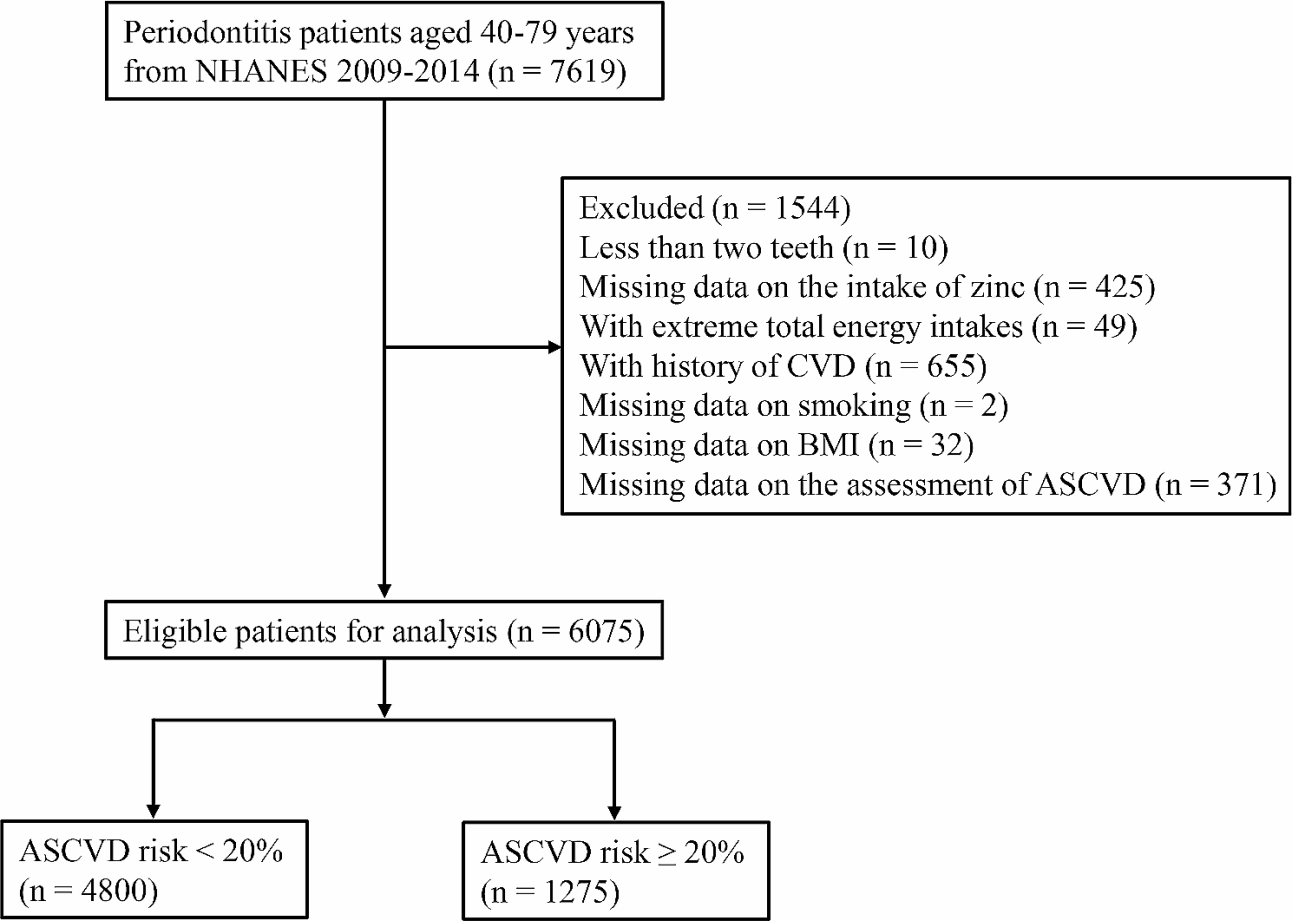
The flowchart of selecting patients

In this study, 7,619 periodontitis patients with age of 40–79 years were extracted from the NHANES database. Of these, 1,544 patients were excluded due to less than two teeth (*n* = 10), missing data on the intake of zinc (*n* = 425), with extreme total energy intakes (*n* = 49), with history of CVD (*n* = 655), missing data on smoking (*n* = 2), missing data on BMI (*n* = 32), and missing data on the assessment of ASCVD (*n* = 371). Finally, 6,075 patients were included for analysis, with 1,275 patients assessed with low to intermediate risk of ASCVD and 4,000 patients assessed with 10-year ASCVD risk ≥ 20% (Fig. 1). For missing variables, sensitivity analysis showed that there was no significant difference in estimates before versus after imputation (Supplementary Table S1).

The characteristics of eligible patients were shown in Table 1. The mean age was 54.31 (0.18) years, and 48.51% of the patients (*n* = 2961) were male. Significant difference was found in age, gender, race, education level, BMI, smoking, drinking, physical activity, diabetes, diabetes drugs, hypertension drugs, lipid-lowering therapy, HDL-C, HbAlc, decayed teeth, oral hygiene, dental floss, energy, carbohydrate, zinc-RDA, and periodontitis between 10-year ASCVD risk < 20% group and 10-year ASCVD risk ≥ 20% group (*P* < 0.05).

**Table 1:** The characteristics of included patients

Variables	Total (*n* = 6075)	ASCVD risk (low to intermediate) (*n* = 4800)	ASCVD risk (high) (*n* = 1275)	Statistics	*P*
Age, years, Mean (S.E)	54.31 (0.18)	52.09 (0.16)	66.91 (0.30)	t = 42.891	< 0.001
Gender, n (%)				χ² = 180.425	< 0.001
Female	3114 (51.49)	2694 (54.85)	420 (32.40)		
Male	2961 (48.51)	2106 (45.15)	855 (67.60)		
Race, n (%)				χ² = 19.482	< 0.001
Mexican American	944 (6.88)	765 (7.06)	179 (5.84)		
Non-Hispanic Black	1279 (9.69)	873 (8.56)	406 (16.10)		
Non-Hispanic White	2555 (72.33)	2062 (72.77)	493 (69.84)		
Other Hispanic	658 (4.82)	549 (5.01)	109 (3.72)		
Other Race	639 (6.28)	551 (6.60)	88 (4.50)		
Education level, n (%)				χ² = 6.482	0.014
Above or college graduate	3359 (64.97)	2740 (65.87)	619 (59.84)		
Below college graduate	2716 (35.03)	2060 (34.13)	656 (40.16)		
PIR, n (%)				χ² = 0.153	0.842
< 1	933 (8.95)	747 (8.87)	186 (9.43)		
≥ 1	4649 (84.78)	3658 (84.88)	991 (84.20)		
Unknown	493 (6.27)	395 (6.25)	98 (6.38)		
Marital status, n (%)				χ² = 2.779	0.102
Married	3689 (66.04)	2935 (66.46)	754 (63.61)		
No married	2386 (33.96)	1865 (33.54)	521 (36.39)		
BMI, n (%)				χ² = 4.754	0.014
Underweight/normal	1494 (25.38)	1208 (26.02)	286 (21.79)		
Overweight	2200 (37.19)	1725 (37.32)	475 (36.46)		
Obesity	2381 (37.43)	1867 (36.67)	514 (41.75)		
Smoking, n (%)				χ² = 19.730	< 0.001
No	5007 (83.78)	4038 (84.80)	969 (78.01)		
Yes	1068 (16.22)	762 (15.20)	306 (21.99)		
Drinking, n (%)				χ² = 5.594	0.002
< once/week	1676 (28.84)	1356 (29.27)	320 (26.43)		
≥ once/week	1950 (39.62)	1559 (40.45)	391 (34.89)		
Never drinking	1527 (18.76)	1214 (18.10)	313 (22.48)		
Unknown	922 (12.78)	671 (12.18)	251 (16.21)		
Physical activity, n (%)				χ² = 7.912	0.001
< 450 MET·min/week	694 (11.05)	536 (10.86)	158 (12.14)		
≥ 450 MET·min/week	3890 (67.23)	3152 (68.41)	738 (60.50)		
Unknown	1491 (21.73)	1112 (20.73)	379 (27.37)		
Sedentary time, n (%)				χ² = 1.726	0.186
< 7.5 h	3933 (60.04)	3091 (59.48)	842 (63.24)		
≥ 7.5 h	2118 (39.61)	1691 (40.17)	427 (36.40)		
Unknown	24 (0.35)	18 (0.35)	6 (0.36)		
Diabetes, n (%)				χ² = 199.537	< 0.001
No	2531 (44.99)	2291 (49.58)	240 (18.93)		
Yes	3544 (55.01)	2509 (50.42)	1035 (81.07)		
Diabetes drugs, n (%)				χ² = 83.411	< 0.001
No	5335 (90.83)	4358 (92.86)	977 (79.32)		
Yes	740 (9.17)	442 (7.14)	298 (20.68)		
Hypertension drugs, n (%)				χ² = 112.170	< 0.001
No	4046 (69.40)	3496 (73.63)	550 (45.40)		
Yes	2029 (30.60)	1304 (26.37)	725 (54.60)		
Lipid-lowering therapy, n (%)				χ² = 61.388	< 0.001
No	4772 (78.13)	3959 (80.81)	813 (62.88)		
Yes	1303 (21.87)	841 (19.19)	462 (37.12)		
Nonsteroidal drugs, n (%)				χ² = 0.680	0.414
No	5710 (94.61)	4519 (94.74)	1191 (93.91)		
Yes	365 (5.39)	281 (5.26)	84 (6.09)		
Anti-infective drugs, n (%)				χ² = 2.222	0.143
No	5813 (95.11)	4583 (94.89)	1230 (96.33)		
Yes	262 (4.89)	217 (5.11)	45 (3.67)		
TC, mg/dL, Mean (S.E)	203.28 (0.77)	203.61 (0.81)	201.37 (1.68)	t = -1.298	0.200
HDL-C, mg/dL, Mean (S.E)	54.63 (0.38)	55.20 (0.41)	51.37 (0.59)	t = -6.004	< 0.001
HbAlc, %, Mean (S.E)	5.73 (0.02)	5.66 (0.02)	6.13 (0.04)	t = 10.525	< 0.001
Serum vitamin D, n (%)				χ² = 0.996	0.323
< 50 nmol/L	1703 (19.81)	1325 (19.57)	378 (21.16)		
≥ 50 nmol/L	4372 (80.19)	3475 (80.43)	897 (78.84)		
WBC, 1000 cells/uL, Mean (S.E)	6.95 (0.05)	6.94 (0.05)	7.05 (0.09)	t = 1.359	0.181
Decayed teeth, n (%)				χ² = 4.627	0.013
No	21 (0.31)	14 (0.27)	7 (0.52)		
Unknown	4268 (76.62)	3437 (77.38)	831 (72.33)		
Yes	1786 (23.06)	1349 (22.34)	437 (27.15)		
Oral hygiene, n (%)				χ² = 6.079	0.004
Yes	2684 (34.74)	2028 (33.72)	656 (40.52)		
Unknown	3381 (65.15)	2762 (66.15)	619 (59.48)		
No	10 (0.11)	10 (0.13)	0 (0.00)		
Dental floss, n (%)				χ² = 6.231	0.007
No	1801 (25.11)	1319 (24.03)	482 (31.26)		
Unknown	44 (0.41)	32 (0.41)	12 (0.42)		
Yes	4230 (74.48)	3449 (75.56)	781 (68.32)		
Energy, kcal, Mean (S.E)	2147.37 (18.35)	2162.82 (20.74)	2059.65 (33.63)	t = -2.605	0.012
Carbohydrate, gm, Mean (S.E)	252.88 (2.28)	254.62 (2.58)	243.01 (4.07)	t = -2.405	0.020
Vitamin C, mg, Mean (S.E)	84.16 (2.08)	84.11 (2.21)	84.39 (3.55)	t = 0.076	0.940
Zinc-RDA, n (%)				χ² = 15.939	< 0.001
No	2861 (43.15)	2169 (42.02)	692 (49.61)		
Yes	3214 (56.85)	2631 (57.98)	583 (50.39)		
Periodontitis, n (%)				χ² = 104.060	< 0.001
No	2424 (48.77)	2129 (52.04)	295 (30.21)		
Yes	3651 (51.23)	2671 (47.96)	980 (69.79)		
Zinc, mg, Mean (S.E)	11.48 (0.12)	11.51 (0.13)	11.35 (0.29)	t = -0.541	0.591

Mean (S.E), mean (standard error); PIR, poverty-to-income ratio; BMI, body mass index; MET, metabolic equivalent of task; TC, total cholesterol; HDL-C, high density lipoprotein cholesterol; HbAlc, glycated hemoglobin; WBC, white blood cell count; RDA, recommended dietary allowances

### Association between zinc-RDA or periodontitis and 10-year ASCVD risk ≥ 20%

Supplementary Table S2 shows that education level, BMI, drinking, lipid lowering therapy, dental floss, energy, and carbohydrate were identified as confounders. In the unadjusted model, we found that zinc-RDA was associated with the decreased odds of 10-year ASCVD risk ≥ 20% (OR = 0.74, 95%CI: 0.63–0.86). Periodontitis was associated with the high odds of 10-year ASCVD risk ≥ 20% (OR = 2.51, 95%CI: 2.09-3.00). After adjusting the confounders, we found the similar associations (zinc-RDA: OR = 0.79, 95%CI: 0.66–0.94; periodontitis: OR = 2.49, 95%CI: 2.06–3.01). Further adjusting periodontitis based on Model 2, we found the association between zinc-RDA and the lower odds of 10-year ASCVD risk ≥ 20% (OR = 0.82, 95%CI: 0.69–0.98). Further adjusting zinc-RDA based on Model 2, we found the association between periodontitis and the higher odds of 10-year ASCVD risk ≥ 20% (OR = 2.47, 95%CI: 2.04-3.00). The results were summarized in Table 2.

**Table 2:** Association between zinc-RDA or periodontitis and 10-year ASCVD risk ≥ 20%

Variables	Model 1		Model 2		Model 3	
	OR (95% CI)	*P*	OR (95% CI)	*P*	OR (95% CI)	*P*
Zinc-RDA						
No	Ref		Ref		Ref	
Yes	0.74 (0.63–0.86)	< 0.001	0.79 (0.66–0.94)	0.012	0.82 (0.69–0.98)^*^	0.032
**Periodontitis**						
No	Ref		Ref		Ref	
Yes	2.51 (2.09-3.00)	< 0.001	2.49 (2.06–3.01)	< 0.001	2.47 (2.04-3.00)^#^	< 0.001

OR, odds ratio; CI, confidence interval; Ref, reference; RDA, recommended dietary allowances; ASCVD, atherosclerotic cardiovascular disease

Zinc-RDA no as ref group means individuals whose dietary zinc intake did not reach the RDA were used as the reference group

Periodontitis no as ref group means individuals without periodontitis were used as the reference group

Model 1, unadjusted model;

Model 2, adjusting education level, BMI, drinking, lipid lowering therapy, dental floss, energy, and carbohydrate;

Model 3, ^*^adjusting periodontitis based on Model 2; ^#^adjusting zinc-RDA based on Model 2

### The effect of zinc-RDA on the association between periodontitis and 10-year ASCVD risk ≥ 20%

**Fig. 2:**
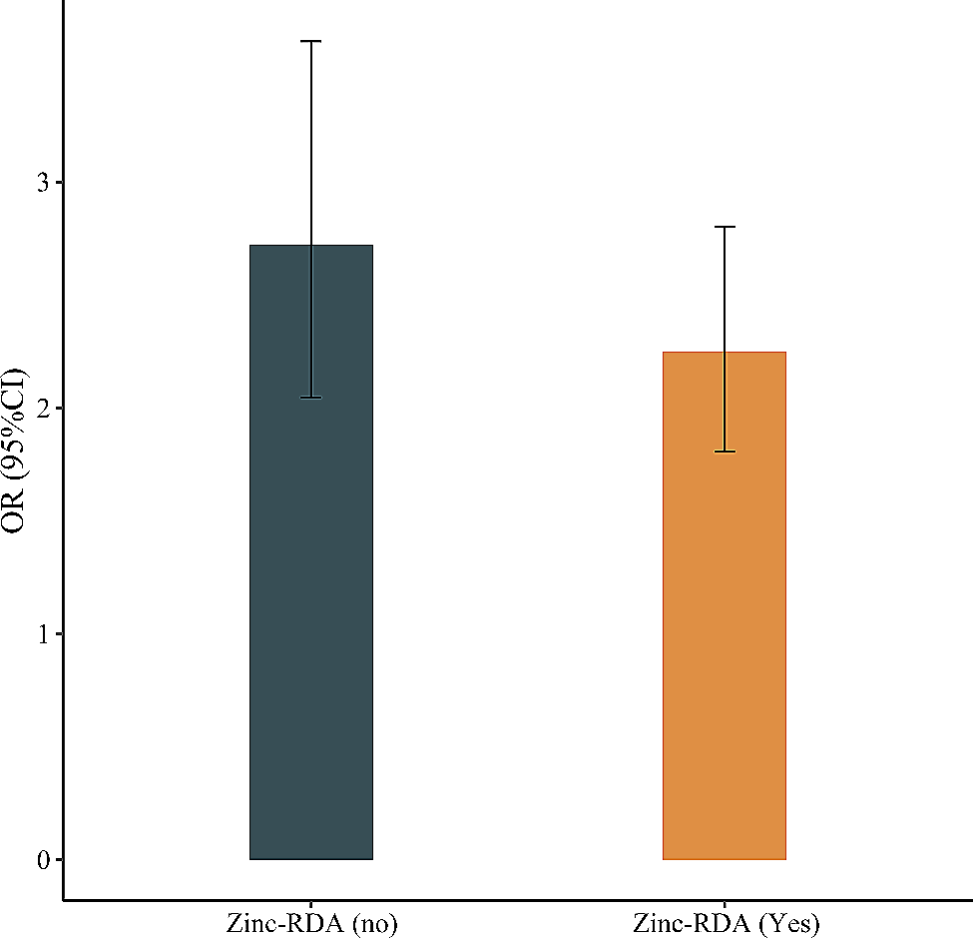
The odds of 10-year ASCVD risk ≥ 20% in periodontitis patients with or without recommended level of zinc intake

In patients without zinc-RDA, we found the higher odds of 10-year ASCVD risk ≥ 20% in periodontitis patients after adjusting education level, BMI, drinking, lipid lowering therapy, dental floss, energy, and carbohydrate (OR = 2.72, 95%CI: 2.05–3.62). In patients with zinc-RDA, the odds were decreased a little (OR = 2.25, 95%CI: 1.81–2.80) (Fig. 2). The results were summarized in Table 3.

**Table 3:** The effect of zinc-RDA on the association between periodontitis and 10-year ASCVD risk ≥ 20%

Variables	Model 1		Model 2	
	OR (95% CI)	*P*	OR (95% CI)	*P*
Zinc-RDA (no)				
Periodontitis				
No	Ref		Ref	
Yes	2.72 (2.08–3.55)	< 0.001	2.72 (2.05–3.62)	< 0.001
Zinc-RDA (yes)				
Periodontitis				
No	Ref		Ref	
Yes	2.30 (1.85–2.87)	< 0.001	2.25 (1.81–2.80)	< 0.001

OR, odds ratio; CI, confidence interval; RDA, recommended dietary allowances; ASCVD, atherosclerotic cardiovascular disease

Model 1, unadjusted model;

Model 2, adjusting education level, BMI, drinking, lipid lowering therapy, dental floss, energy, and carbohydrate

### Subgroup analysis for the association between zinc-RDA or periodontitis and 10-year ASCVD risk ≥ 20%

Zinc-RDA was found to be associated with the lower odds of 10-year ASCVD risk ≥ 20% in patients with age ≥ 60 years (OR = 0.61, 95%CI: 0.47–0.78), with age < 60 years (OR = 0.74, 95%CI: 0.57–0.97), with education level above or college graduate (OR = 0.78, 95%CI: 0.61–0.98), without lipid lowering therapy (OR = 0.79, 95%CI: 0.62–0.99), and with use of dental floss (OR = 0.79, 95%CI: 0.64–0.99). Periodontitis was found to be associated with the higher odds of 10-year ASCVD risk ≥ 20% in patients with age < 60 years or ≥ 60 years, with gender of male or female, with or without obesity, with education level of above or college graduate and below college graduate, with or without lipid lowering therapy, and with or without use of dental floss (all *P* < 0.05). The results were summarized in Supplementary Table S3.

**Subgroup analysis for the effect of zinc-RDA on the association between periodontitis and 10-year ASCVD risk ≥ 20%**

Table 4 ASCVD risk ≥ 20% was decreased in periodontitis patients with zinc-RDA compared to those without zinc-RDA in patients with age ≥ 60 years [OR (95%CI): 3.92 (1.74–8.82) vs. 4.71 (2.52–8.79)] (Supplementary Fig. 1A), with age < 60 years [OR (95%CI): 1.45 (1.07–1.97) vs. 1.86 (1.30–2.66)] (Supplementary Fig. 1B), in male [OR (95%CI): 2.23 (1.51–3.29) vs. 2.81 (1.92–4.11)] (Supplementary Fig. 2A), in female [OR (95%CI): 1.51 (1.03–2.23) vs. 1.85 (1.28–2.67)] (Supplementary Fig. 2B), with obesity [OR (95%CI): 2.39 (1.76–3.25) vs. 3.12 (2.27–4.28)] (Supplementary Fig. 3A), without obesity [OR (95%CI): 1.99 (1.31–3.04) vs. 2.32 (1.36–3.96)] (Supplementary Fig. 3B), with education level of above or college graduate [OR (95%CI): 2.54 (1.82–3.53) vs. 3.11 (2.14–4.52)] (Supplementary Fig. 4A), with education level of below college graduate [OR (95%CI): 1.57 (1.13–2.18) vs. 2.13 (1.45–3.11)] (Supplementary Fig. 4B), with lipid lowering therapy [OR (95%CI): 2.05 (1.25–3.38) vs. 2.78 (1.70–4.56)] (Supplementary Fig. 5A), without lipid lowering therapy [OR (95%CI): 2.42 (1.85–3.15) vs. 2.76 (1.99–3.83)] (Supplementary Fig. 5B), with use of dental floss [OR (95%CI): 2.27 (1.70–3.02) vs. 2.85 (2.02–4.02)] (Supplementary Fig. 6A), and without use of dental floss [OR (95%CI): 2.12 (1.32–3.42) vs. 2.50 (1.31–4.76)] (Supplementary Fig. 6B).

**Table 4:** Subgroup analysis for the effect of zinc-RDA on the association between periodontitis and 10-year ASCVD risk ≥ 20%

Subgroups	Zinc-RDA (no)				Zinc-RDA (yes)			
	Periodontitis (no)		Periodontitis (yes)		Periodontitis (no)		Periodontitis (yes)	
	OR (95%CI)	*P*	OR (95%CI)	*P*	OR (95%CI)	*P*	OR (95% CI)	*P*
Age ≥ 60 years	Ref		4.71 (2.52–8.79)	< 0.001	Ref		3.92 (1.74–8.82)	0.002
Age < 60 years	Ref		1.86 (1.30–2.66)	0.002	Ref		1.45 (1.07–1.97)	0.022
Male	Ref		2.81 (1.92–4.11)	< 0.001	Ref		2.23 (1.51–3.29)	< 0.001
Female	Ref		1.85 (1.28–2.67)	0.002	Ref		1.51 (1.03–2.23)	0.043
Obesity (yes)	Ref		3.12 (2.27–4.28)	< 0.001	Ref		2.39 (1.76–3.25)	< 0.001
Obesity (no)	Ref		2.32 (1.36–3.96)	0.004	Ref		1.99 (1.31–3.04)	0.003
Education level (above or college graduate)	Ref		3.11 (2.14–4.52)	< 0.001	Ref		2.54 (1.82–3.53)	< 0.001
Education level (below college graduate)	Ref		2.13 (1.45–3.11)	< 0.001	Ref		1.57 (1.13–2.18)	0.010
Lipid lowering therapy (yes)	Ref		2.78 (1.70–4.56)	< 0.001	Ref		2.05 (1.25–3.38)	0.008
Lipid lowering therapy (no)	Ref		2.76 (1.99–3.83)	< 0.001	Ref		2.42 (1.85–3.15)	< 0.001
Dental floss (yes)	Ref		2.85 (2.02–4.02)	< 0.001	Ref		2.27 (1.70–3.02)	< 0.001
Dental floss (no)	Ref		2.50 (1.31–4.76)	0.008	Ref		2.12 (1.32–3.42)	0.004

OR, odds ratio; CI, confidence intervals; RDA, recommended dietary allowances; ASCVD, atherosclerotic cardiovascular disease

## Discussion

In this study, we found that zinc intake reaching RDA was associated with the lower odds of 10-year ASCVD risk ≥ 20% and periodontitis was associated with the higher odds of 10-year ASCVD risk ≥ 20%. We also found that the odds of 10-year ASCVD risk ≥ 20% was decreased in periodontitis patients with zinc intake reaching RDA. The similar relationship was also found in periodontitis patients with age ≥ 60 years or < 60 years, with gender of male or female, with or without obesity, with education level of below, above or in college graduate, with or without lipid lowering therapy, and with or without use of dental floss.

Previous studies have reported that periodontitis imparts increased risk for future ASCVD [4, 20]. In this study, we also found that periodontitis was associated with the high odds of 10-year ASCVD risk ≥ 20%. The micronutrient zinc is essential to all living organisms and involved in many biochemical pathways in human cells [8]. Inadequate intake of zinc is associated with many pathological conditions, including CVDs [8]. Atherosclerosis is a main reason for CVD [21]. A key feature of atherosclerosis is the increase of oxidative stress, which leads to endothelial damage, NF-κB-related signaling disorder, and the oxidative modification of low-density lipoprotein [8]. Zinc participates in all of these aspects through its antioxidant and anti-inflammatory functions [22]. This present study showed that zinc intake reaching the recommend level was associated with the lower odds of 10-year ASCVD risk ≥ 20%. In addition, we also found that adequate intake of zinc (reaching the recommend level) improved the effect of periodontitis on the ASCVD risk. The inflammatory response elicited by periodontal pathogens triggers a systemic inflammatory cascade, contributing to endothelial dysfunction and atherosclerosis [23]. Moreover, periodontitis-induced oxidative stress plays a pivotal role in endothelial dysfunction and vascular damage, thereby promoting atherosclerosis [24]. Reactive oxygen species generated in response to periodontal infection overwhelm endogenous antioxidant defenses, leading to oxidative damage to lipids, proteins, and DNA [25]. Evidence has reported a potential regulatory relationship between zinc and periodontitis [26]. High intake of zinc could decrease the odds of periodontitis and improve the microenvironment of periodontitis by antioxidant and anti-inflammatory activities [27], thereby decreasing the odds of further ASCVD risk.

Previous studies have reported age, gender, and education level as the influencing factors for periodontitis [28–30]. Our results showed that adequate intake of zinc was associated the lower odds of 10-year ASCVD risk ≥ 20% in periodontitis patients with different age, gender, and education levels, indicating the potential universality of the beneficial effects of zinc supplementation in individuals with periodontitis who are at risk for ASCVD. Given the well-established age-related decline in zinc intake and increased susceptibility to both periodontitis and ASCVD with advancing age, ensuring adequate zinc intake may represent a variable strategy for preserving cardiovascular health in older adults [31, 32]. Gender-specific differences in the prevalence and severity of both periodontitis and ASCVD have been observed [33, 34]. Our findings suggest that the beneficial effects of dietary zinc may transcend gender differences. The finding in subgroup of different education levels emphasizes the importance of targeted interventions to improve dietary zinc intake, particularly in populations with lower socioeconomic status, as a means of reducing ASCVD risk burden. Obesity is a risk factor for periodontitis, and adipokines (found in obesity) may induce inflammation and atherosclerosis [35, 36]. Our results suggested that the odds of 10-year ASCVD risk ≥ 20% was decreased by adequate intake of zinc in periodontitis patients with or without obesity.

Periodontal pathogenic bacteria and pro-inflammatory cytokines caused by periodontitis can lead to lipid metabolism disorders [37]. In addition, hyperlipidemia and lipid peroxidation stimulate pro-inflammatory cytokines, leading to oxidative stress and delayed wound healing, making individuals susceptible to periodontitis [37]. Considering the antioxidant and anti-inflammatory property of zinc, we performed subgroup on lipid lowering therapy. The similar results were found in patients with or without lipid lowering therapy. Evidence has shown that interdental cleaning devices, including dental floss, could decrease dental plaque, optimize the oral hygiene, and support gingival health [38, 39]. Our study found the lower odds of ASCVD after adequate zinc intake in periodontitis patients with or without use of dental floss. The finding suggest that the protective effect of zinc may operate independently of oral hygiene practices, highlighting the multifactorial nature of the relationship between periodontitis and ASCVD. While the role of oral hygiene practices in ASCVD prevention has been extensively studied, our findings suggest that optimizing nutrient intake, such as zinc, may complement existing periodontal interventions in mitigating cardiovascular risk.

This study uses a nationally representative samples from the NHANES database to explore the regulatory effect of zinc intake on the association between periodontitis and the odds of ASCVD risk ≥ 20%, which may provide guidance for the management of periodontitis to reduce the disease burden. However, there are several limitations in this study. First, due to the cross-sectional study design, it is unable to infer causality. However, this study evaluates the 10-year risk of ASCVD based on the score recommended by ACC/AHA and excludes patients with a history of CVD, which has certain value for assessment of risk of CVD. Second, zinc intake is from diet and dietary supplements, which may not reflect a person’s daily diet.

## Conclusion

This study found the regulatory effect of adequate zinc intake on the association between periodontitis and ASCVD, which provided insight to decrease the risk of ASCVD for patients with periodontitis.

### Electronic supplementary material

Below is the link to the electronic supplementary material.

Supplementary Material 1

Supplementary Material 2

Supplementary Material 3

Supplementary Material 4

Supplementary Material 5

Supplementary Material 6

Supplementary Material 7

Supplementary Material 8

Supplementary Material 9

## Data Availability

The datasets generated during and/or analyzed during the current study are available in the NHANES database, https://www.cdc.gov/nchs/nhanes/index.htm.

## Abbreviations

ASCVD: Atherosclerotic cardiovascular disease
CVD: Cardiovascular diseases
ACC: American College of Cardiology
AHA: American Heart Association
NHANES: National Health and Nutrition Examination Survey
BMI: Body mass index
MEC: Mobile Examination Center
RDA: Recommended dietary allowances
PIR: Poverty-to-income ratio
TC: Total cholesterol
HDL-C: High density lipoprotein cholesterol
HbAlc: Glycated hemoglobin
WBC: White blood cell count
MET: Metabolic equivalent of task
FBG: Fasting blood glucose
S.E: Standard error
OR: Odds ratio
CI: Confidence interval
