# Supplementary material for: The regulatory effect of zinc on the association between periodontitis and atherosclerotic cardiovascular disease: a cross-sectional study based on the National Health and Nutrition Examination Survey
Source: BMC Oral Health. 2024 Jun 18;24:703. doi: 10.1186/s12903-024-04473-6 (PMC11184828; doi:10.1186/s12903-024-04473-6)
Supplement: Supplementary file 1 — Supplementary Material 1 [file 12903_2024_4473_MOESM1_ESM.docx]

**Supplementary figure 1** The odds of 10-year ASCVD risk ≥ 20% in periodontitis patients with or without recommended level of zinc intake at age ≥ 60 years (A) and < 60 years (B).

**Supplementary figure 2** The odds of 10-year ASCVD risk ≥ 20% in periodontitis patients with or without recommended level of zinc intake in male (A) and in female (B).

**Supplementary figure 3** The odds of 10-year ASCVD risk ≥ 20% in periodontitis patients with or without recommended level of zinc intake under condition of obesity (A) and without obesity (B).

**Supplementary figure 4** The odds of 10-year ASCVD risk ≥ 20% in periodontitis patients with or without recommended level of zinc intake under education level of above or college graduate (A) and below college graduate (B).

**Supplementary figure 5** The odds of 10-year ASCVD risk ≥ 20% in periodontitis patients with or without recommended level of zinc intake under condition of lipid lowering therapy (A) and without lipid lowering therapy (B).

**Supplementary figure 6** The odds of 10-year ASCVD risk ≥ 20% in periodontitis patients with or without recommended level of zinc intake under condition of using dental floss (A) and without using dental floss (B).
