# Supplementary material for: The regulatory effect of zinc on the association between periodontitis and atherosclerotic cardiovascular disease: a cross-sectional study based on the National Health and Nutrition Examination Survey
Source: BMC Oral Health. 2024 Jun 18;24:703. doi: 10.1186/s12903-024-04473-6 (PMC11184828; doi:10.1186/s12903-024-04473-6)
Supplement: Supplementary file 2 — Supplementary Material 2 [file 12903_2024_4473_MOESM2_ESM.docx]

**Supplementary table S1 Sensitivity analysis for imputation**

| **Variables** | **Before (n = 6075)** | **After (n = 6075)** | **Statistics** | ***P*** |
| --- | --- | --- | --- | --- |
| Education level, n (%) |  |  | χ² = 1.701 | 0.198 |
| Above or college graduate | 3357 (64.98) | 3359 (64.97) |  |  |
| Below college graduate | 2713 (35.02) | 2716 (35.03) |  |  |
| Marital status, n (%) |  |  | χ² = 1.269 | 0.266 |
| Married | 3685 (66.02) | 3689 (66.04) |  |  |
| No married | 2385 (33.98) | 2386 (33.96) |  |  |
| HbAlc, %, Mean (S.E) | 5.73 (0.02) | 5.73 (0.02) | t = -0.055 | 0.957 |
| WBC, 1000 cells/uL, Mean (S.E) | 6.95 (0.05) | 6.95 (0.05) | t = -1.015 | 0.315 |

Mean (S.E), mean (standard error); HbAlc, glycated hemoglobin; WBC, white blood cell count.

**Supplementary table S2 Selection of confounders by univariate logistic regression model**

| **Variables** | **OR (95% CI)** | ***P*** |
| --- | --- | --- |
| Education level |  |  |
| Above or college graduate | Ref |  |
| Below college graduate | 1.30 (1.06-1.58) | 0.014 |
| PIR |  |  |
| < 1 | Ref |  |
| ≥ 1 | 0.93 (0.72-1.22) | 0.614 |
| Unknown | 0.96 (0.70-1.32) | 0.800 |
| Marital status |  |  |
| Married | Ref |  |
| No married | 1.13 (0.98-1.31) | 0.102 |
| BMI |  |  |
| Underweight/Normal | Ref |  |
| Overweight | 1.17 (0.99-1.37) | 0.071 |
| Obesity | 1.36 (1.11-1.67) | 0.005 |
| Drinking |  |  |
| < once/week | Ref |  |
| ≥ once/week | 0.96 (0.73-1.24) | 0.734 |
| Never drinking | 1.38 (1.05-1.81) | 0.028 |
| Unknown | 1.47 (1.11-1.95) | 0.009 |
| Physical activity |  |  |
| < 450 MET·min/week | Ref |  |
| ≥ 450 MET·min/week | 0.79 (0.61-1.03) | 0.085 |
| Unknown | 1.18 (0.88-1.58) | 0.265 |
| Sedentary time |  |  |
| < 7.5h | Ref |  |
| ≥ 7.5h | 0.85 (0.71-1.03) | 0.098 |
| Unknown | 0.98 (0.29-3.27) | 0.974 |
| Lipid lowering therapy |  |  |
| No | Ref |  |
| Yes | 2.49 (1.97-3.14) | < 0.001 |
| Nonsteroidal drug |  |  |
| No | Ref |  |
| Yes | 1.17 (0.81-1.68) | 0.414 |
| Anti-infective drugs |  |  |
| No | Ref |  |
| Yes | 0.71 (0.45-1.12) | 0.145 |
| TC | 1.00 (1.00-1.00) | 0.219 |
| Serum vitamin D |  |  |
| < 50 nmol/L | Ref |  |
| ≥ 50 nmol/L | 0.91 (0.75-1.10) | 0.324 |
| WBC | 1.03 (0.99-1.06) | 0.183 |
| Decayed teeth |  |  |
| No | Ref |  |
| Unknown | 0.49 (0.13-1.81) | 0.291 |
| Yes | 0.64 (0.17-2.38) | 0.509 |
| Oral hygiene |  |  |
| Yes | Ref |  |
| Unknown | 0.75 (0.62-0.90) | 0.004 |
| No | -- | - |
| Dental floss |  |  |
| No | Ref |  |
| Unknown | 0.80 (0.31-2.02) | 0.636 |
| Yes | 0.70 (0.54-0.90) | 0.008 |
| Energy | 1.00 (1.00-1.00) | 0.013 |
| Carbohydrate | 1.00 (1.00-1.00) | 0.020 |
| Vitamin C | 1.00 (1.00-1.00) | 0.940 |

OR, odds ratio; CI, confidence interval; PIR, poverty-to-income ratio; BMI, body mass index; MET, metabolic equivalent of task; TC, total cholesterol; WBC, white blood cell count.

**Supplementary table S3 Subgroup analysis for the association between zinc-RDA or periodontitis and 10-year ASCVD risk ≥ 20%**

| **Subgroups** | **Zinc-RDA** | | | | **Periodontitis** | | | |
| --- | --- | --- | --- | --- | --- | --- | --- | --- |
|  | **No** | | **Yes** | | **No** | | **Yes** | |
|  | **OR (95% CI)** | ***P*** | **OR (95% CI)** | ***P*** | **OR (95% CI)** | ***P*** | **OR (95% CI)** | ***P*** |
| Age ≥ 60 years | Ref | | 0.61 (0.47-0.78) | < 0.001 | Ref | | 4.28 (2.60-7.04) | < 0.001 |
| Age < 60 years | Ref | | 0.74 (0.57-0.97) | 0.036 | Ref | | 1.63 (1.30-2.04) | < 0.001 |
| Male | Ref | | 1.05 (0.83-1.32) | 0.714 | Ref | | 2.51 (1.87-3.38) | < 0.001 |
| Female | Ref | | 1.01 (0.76-1.35) | 0.928 | Ref | | 1.66 (1.29-2.15) | < 0.001 |
| Obesity (yes) | Ref | | 0.78 (0.59-1.02) | 0.075 | Ref | | 2.72 (2.12-3.48) | < 0.001 |
| Obesity (no) | Ref | | 0.89 (0.67-1.19) | 0.448 | Ref | | 2.16 (1.49-3.14) | < 0.001 |
| Education level (above or college graduate) | Ref | | 0.78 (0.61-0.98) | 0.040 | Ref | | 2.82 (2.15-3.70) | < 0.001 |
| Education level (below college graduate) | Ref | | 0.87 (0.64-1.18) | 0.383 | Ref | | 1.80 (1.36-2.39) | < 0.001 |
| Lipid lowering therapy (yes) | Ref | | 0.90 (0.64-1.27) | 0.553 | Ref | | 2.29 (1.57-3.35) | < 0.001 |
| Lipid lowering therapy (no) | Ref | | 0.79 (0.62-0.99) | 0.049 | Ref | | 2.57 (2.05-3.23) | < 0.001 |
| Dental floss (yes) | Ref | | 0.79 (0.64-0.99) | 0.045 | Ref | | 2.50 (1.92-3.26) | < 0.001 |
| Dental floss (no) | Ref | | 0.86 (0.64-1.17) | 0.342 | Ref | | 2.25 (1.45-3.51) | 0.001 |

OR, odds ratio; CI, confidence intervals; RDA, recommended dietary allowances; ASCVD, atherosclerotic cardiovascular disease.
