# Supplementary material for: The regulatory effect of zinc on the association between periodontitis and atherosclerotic cardiovascular disease: a cross-sectional study based on the National Health and Nutrition Examination Survey
Source: BMC Oral Health. 2024 Jun 18;24:703. doi: 10.1186/s12903-024-04473-6 (PMC11184828; doi:10.1186/s12903-024-04473-6)
Supplement: Supplementary file 9 — Supplementary Material 9 [file 12903_2024_4473_MOESM9_ESM.pdf]

STROBE Statement—checklist of items that should be included in reports of observational studies

	Item No.	Recommendation	Page No.	Relevant text from manuscript
**Title and abstract**	1	(*a*) Indicate the study's design with a commonly used term in the title or the abstract	Page 1	Line 1-3
		(*b*) Provide in the abstract an informative and balanced summary of what was done and what was found	Page 2-3	Line 15-42
**Introduction**				
Background/rationale	2	Explain the scientific background and rationale for the investigation being reported	Page 4-5	Line 44-67
Objectives	3	State specific objectives, including any prespecified hypotheses	Page 5	Line 67-71
**Methods**				
Study design	4	Present key elements of study design early in the paper	Page 5	Line 74-76
Setting	5	Describe the setting, locations, and relevant dates, including periods of recruitment, exposure, follow-up, and data collection	Page 5-6	Line 77-90
Participants	6	(*a*) *Cohort study*—Give the eligibility criteria, and the sources and methods of selection of participants. Describe methods of follow-up<br>*Case-control study*—Give the eligibility criteria, and the sources and methods of case ascertainment and control selection. Give the rationale for the choice of cases and controls<br>*Cross-sectional study*—Give the eligibility criteria, and the sources and methods of selection of participants	Page 6	Line 90-97
		(*b*) *Cohort study*—For matched studies, give matching criteria and number of exposed and unexposed<br>*Case-control study*—For matched studies, give matching criteria and the number of controls per case		
Variables	7	Clearly define all outcomes, exposures, predictors, potential confounders, and effect modifiers. Give diagnostic criteria, if applicable	Page 6-8	Line 98-149
Data sources/ measurement	8*	For each variable of interest, give sources of data and details of methods of assessment (measurement). Describe comparability of assessment methods if there is more than one group	Page 6-8	Line 98-149
Bias	9	Describe any efforts to address potential sources of bias	Page 9	Line 160-161
Study size	10	Explain how the study size was arrived at	Page 6	Line 89-97

Continued on next page

Quantitative variables	11	Explain how quantitative variables were handled in the analyses. If applicable, describe which groupings were chosen and why	Page 8-9	Line 152-155
Statistical methods	12	(*a*) Describe all statistical methods, including those used to control for confounding	Page 8-9	Line 151-172
		(*b*) Describe any methods used to examine subgroups and interactions	Page 9	Line 168-169
		(*c*) Explain how missing data were addressed	Page 9	Line 156-159
		(*d*) *Cohort study*—If applicable, explain how loss to follow-up was addressed<br>*Case-control study*—If applicable, explain how matching of cases and controls was addressed<br>*Cross-sectional study*—If applicable, describe analytical methods taking account of sampling strategy	Page 8-9	Line 151-172
		(*e*) Describe any sensitivity analyses	Page 9	Line 158-159
**Results**				
Participants	13*	(a) Report numbers of individuals at each stage of study—eg numbers potentially eligible, examined for eligibility, confirmed eligible, included in the study, completing follow-up, and analysed	Page 10	Line 176-183
		(b) Give reasons for non-participation at each stage		
		(c) Consider use of a flow diagram	Page 10	Line 183
Descriptive data	14*	(a) Give characteristics of study participants (eg demographic, clinical, social) and information on exposures and potential confounders	Page 10	Line 181-192
		(b) Indicate number of participants with missing data for each variable of interest	Page 10	Line 183-185
		(c) *Cohort study*—Summarise follow-up time (eg, average and total amount)		
Outcome data	15*	*Cohort study*—Report numbers of outcome events or summary measures over time		
		Case-control study—Report numbers in each exposure category, or summary measures of exposure		
		Cross-sectional study—Report numbers of outcome events or summary measures	Page 10	Line 181-182
Main results	16	(*a*) Give unadjusted estimates and, if applicable, confounder-adjusted estimates and their precision (eg, 95% confidence interval). Make clear which confounders were adjusted for and why they were included	Page 10-11	Line 194-214
		(*b*) Report category boundaries when continuous variables were categorized	Page 26-28	Line 494-497
		(*c*) If relevant, consider translating estimates of relative risk into absolute risk for a meaningful time period		

Continued on next page

Other analyses	17	Report other analyses done—eg analyses of subgroups and interactions, and sensitivity analyses	Page 11-13	Line 216-248
**Discussion**				
Key results	18	Summarise key results with reference to study objectives	Page 13	Line 251-258
Limitations	19	Discuss limitations of the study, taking into account sources of potential bias or imprecision. Discuss both direction and magnitude of any potential bias	Page 16	Line 319-324
Interpretation	20	Give a cautious overall interpretation of results considering objectives, limitations, multiplicity of analyses, results from similar studies, and other relevant evidence	Page 13-16	Line 259-315
Generalisability	21	Discuss the generalisability (external validity) of the study results	Page 16	Line 272-280
**Other information**				
Funding	22	Give the source of funding and the role of the funders for the present study and, if applicable, for the original study on which the present article is based	Page 18	Line 360-361

*Give information separately for cases and controls in case-control studies and, if applicable, for exposed and unexposed groups in cohort and cross-sectional studies.

Note: An Explanation and Elaboration article discusses each checklist item and gives methodological background and published examples of transparent reporting. The STROBE checklist is best used in conjunction with this article (freely available on the Web sites of PLoS Medicine at http://www.plosmedicine.org/, Annals of Internal Medicine at http://www.annals.org/, and Epidemiology at http://www.epidem.com/). Information on the STROBE Initiative is available at www.strobe-statement.org.
